# Impact of mindfulness and self-compassion on anxiety and depression: The mediating role of resilience

**DOI:** 10.1016/j.ijchp.2021.100229

**Published:** 2021-03-11

**Authors:** Adrián Pérez-Aranda, Javier García-Campayo, Francisco Gude, Juan V. Luciano, Albert Feliu-Soler, Arturo González-Quintela, Yolanda López-del-Hoyo, Jesus Montero-Marin

**Affiliations:** a Aragon Institute for Health Research (IIS Aragón), Miguel Servet University Hospital, Spain; b Faculty of Psychology, Universitat Autònoma de Barcelona, Spain; c AGORA Research Group, Teaching, Research & Innovation Unit, Parc Sanitari Sant Joan de Déu, Spain; d Primary Care Prevention and Health Promotion Research Network, RedIAPP, Spain; e Department of Clinical Epidemiology, Complejo Hospitalario Universitario, Health Research Institute of Santiago de Compostela (IDIS), Spain; f Department of Internal Medicine, Complejo Hospitalario Universitario, Health Research Institute of Santiago de Compostela (IDIS), Spain; g Department of Psychology and Sociology, University of Zaragoza, Spain; h Department of Psychiatry, University of Oxford, Warneford Hospital, United Kingdom

**Keywords:** Mindfulness, Self-compassion, Resilience, Psychopathological symptoms, Ex post fact study, Mindfulness, Autocompasión, Resiliencia, Síntomas psicopatológicos, Estudio ex post fact

## Abstract

*Background/Objective* ‘Third-wave’ psychotherapies have shown effectiveness for treating psychopathological symptoms such as anxiety and depression. There is burgeoning interest in examining how these therapies’ core constructs produce their therapeutic benefits. This study explores the hypothetical mediating effect of resilience in the impact of mindfulness and self-compassion on anxiety and depressive symptoms. *Method*: Cross-sectional study design. The sample consisted of 860 Spanish general population participants. The measures included the Mindful Attention Awareness Scale (MAAS), the Self-Compassion Scale (SCS-12), the Connor-Davidson Resilience Scale (CD-RISC) and the Goldberg Anxiety and Depression Scale (GADS). Bivariate correlations were calculated, and path analysis models were performed. *Results*: Significant correlations were found between the study variables, always in the expected direction (all *p* values <.001). The path analysis models showed significant direct effects of mindfulness and self-compassion on anxiety and depression symptoms, but the only significant indirect effects through resilience were found on depression (MAAS: β = -.05, 95% CI = -.11 to -.02; SCS-12: β = -.06, 95% CI = -.33 to -.07). *Conclusions*: Resilience might partially mediate the effect of mindfulness and self-compassion on depression, but not on anxiety.

## Abstract (es)

*Antecedentes/Objetivo*: Las terapias de “tercera generación” han probado ser eficaces para tratar síntomas psicopatológicos como la ansiedad y la depresión. Hay un interés creciente en examinar cómo los constructos nucleares de estas terapias producen beneficios terapéuticos. Este estudio explora el hipotético efecto mediador de la resiliencia en la relación del mindfulness y la autocompasión sobre los síntomas de ansiedad y depresión. *Método:* Estudio de diseño transversal. Muestra formada por 860 participantes españoles de la población general. Las medidas incluidas fueron: MAAS, SCS-12, CD-RISC y GADS. Se calcularon correlaciones bivariadas y se realizaron modelos de análisis del camino. *Resultados*: Se hallaron correlaciones significativas entre las variables, siempre en la dirección esperada (*p* < 0,001). El modelo de análisis del camino mostró efectos directos significativos de mindfulness y autocompasión sobre síntomas de ansiedad y depresión, pero el único efecto indirecto significativo a través de la resiliencia se encontró en depresión (MAAS: β = -0,05, 95% CI = -0,11 ‒ -0,02; SCS-12: β = -0,06, 95% CI = -0,33 ‒ -0,07). *Conclusiones*: La resiliencia puede mediar parcialmente el efecto de mindfulness y autocompasión sobre la depresión, pero no sobre la ansiedad.

Depression and anxiety are the most common mental disorders in the general population, with a lifetime prevalence of 20.60% in the case of major depressive disorder (Hasin et al., 2018), and a current global prevalence of anxiety disorders of 7.30% (Stein et al., 2017). These conditions, which not seldom are experienced together, often imply a significant loss of quality of life and a certain degree of impairment in different life areas (Khansa et al., 2020).

Among the many different approaches that have been proposed for treating anxiety and depressive symptoms, ‘third-wave’ psychotherapies have been granted much attention in the last two decades. These represent an innovation within cognitive-behavior therapy, as they do not aim at symptom improvement as their only objective and underline themes such as mindfulness, compassion, cognitive fusion, acceptance, and spirituality (Jahoda et al., 2017, Pérez-Aranda et al., 2019). Mindfulness-based interventions, Compassion-Focused Therapy, and Acceptance and Commitment Therapy are some examples of ‘third-wave’ psychotherapies that have proven efficacy for treating different conditions, mainly depressive and anxiety disorders (O’Connor et al., 2018, Pardos-Gascón et al., 2021, Wilson et al., 2019).

The increasing body of evidence of these interventions’ effectiveness has stimulated analyses on how ‘third-wave’ variables may influence the individual’s mental health. In this regard, two possible mediating factors have been studied with growing interest: mindfulness and self-compassion. Mindfulness was defined as the ‘awareness that emerges through paying attention on purpose, in the present moment, and nonjudgmentally to the unfolding of experience’ (Kabat-Zinn, 2003). Although it has been described as a construct that can be trained through practice, mindfulness has also been studied as a trait, and has been observed to play a significant role in emotional self-regulation and depression vulnerability (Guendelman et al., 2017). Other studies have also found a significant mediating effect of mindfulness in psychological wellbeing, as well as depression and anxiety (Pagnini et al., 2019, Takahashi et al., 2019, Takahashi et al., 2020). Self-compassion, on its part, is defined as ‘being touched by and open to one’s own suffering, not avoiding or disconnecting from it, generating the desire to alleviate one’s suffering and to heal oneself with kindness’ (Neff, 2003). It is a strong predictor of reduced depression and trait anxiety, and greater life satisfaction (Van Dam et al., 2011), and different studies have found a mediating effect of self-compassion in anxiety and depression (Mehr and Adams, 2016, Takahashi et al., 2019).

Thus, the relation between mindfulness and self-compassion with anxiety and depression seems to be well documented (Conversano et al., 2020). However, this relation could be mediated by some mechanistic variables, such as resilience. Resilience is defined as a dynamic and flexible process of adaptation to life changes that enables an individual to cope with and recover from stress, and to flourish when faced with adversity (Rutter, 1985), and its protective effect on mental disorders is widely accepted (Southwick & Charney, 2012). Different studies have observed significant associations between resilience and mindfulness (Joyce et al., 2018, Kemper et al., 2015, Montero-Marin et al., 2015). Moreover, there are many studies conducted with clinical and non-clinical samples supporting the mediating role of resilience in the impact of mindfulness on different outcomes related to subjective wellbeing (Bajaj and Pande, 2016, Wang et al., 2016). To our knowledge, the potential mediating role of resilience has not been explored yet in the impact of self-compassion on psychological outcomes, but significant associations between the two variables, along with mindfulness and quality of life, have been reported (Asensio-Martínez et al., 2019, Kemper et al., 2015, Neff and McGehee, 2010, Sünbül and Güneri, 2019).

Considering the abovementioned findings, it seems possible that resilience is somehow promoted by the practice of mindfulness skills and self-compassion. These imply practicing emotion and attention regulation abilities (Diedrich et al., 2014, Hanley et al., 2017) which, in turn, are closely related to the capacity of recovering from stressful situations (Kay, 2016, Mayordomo et al., 2016), to the point that some authors consider ‘emotional resilience’ to be a potential mechanism of mindfulness (Hayes and Feldman, 2006, Polizzi et al., 2018).

In the present study, our main objective was to explore the potential differential mediation role of resilience in the effect of mindfulness and self-compassion on anxiety and depression. Our hypotheses were: (1) mindfulness and self-compassion would have significant direct effects on anxiety and depression, and considering the findings of previous works (Quist-Møller et al., 2018, Van Dam et al., 2011), self-compassion was expected to have a stronger effect than mindfulness; and (2) both mindfulness and self-compassion would have a significant indirect effect on anxiety and depression through the mediating role of resilience.

## Method

### Participants and procedure

The subjects taking part in this cross-sectional study were a subset of the participants in the A Estrada Glycation and Inflammation Study (AEGIS; trial NCT01796184) (Gude et al., 2017). A multistage sampling was carried out in the municipality of A Estrada, Galicia (Spain), with an adult population of 18,897 residents. From November 2012 through March 2015, all subjects were successively convened for 1 day at the Primary Care Center for evaluation. The inclusion criteria were: (1) aged 18 years and older; (2) proficiency in spoken Spanish; and (3) provide informed consent. The exclusion criterion was the presence of severe physical disease. Participants were randomly selected from the Healthcare Registry. First, a computer program (sample function in R) generated a random sample of 3,500 subjects, stratified by age group (in 7 categories, every 10 years). Of these, 639 could not be contacted, 134 lived outside of A Estrada, 19 did not have healthcare coverage, and 84 were deceased. Of the remaining eligible subjects (*N* = 2,624), 394 were excluded due to failure to meet the inclusion criteria, and 714 subjects refused to participate. A total of 1,516 subjects (68%) agreed to participate in the study. In a second stage, two out of three individuals (*N* = 1,010) were invited to participate in the survey, of which 860 subjects completed the questionnaires (58%). This sample size fulfilled the Nunnally and Bernstein (1994) recommendations‒a minimum of 10 participants per variable‒for performing path analytic approximations.

The present study was approved by the Regional Ethics Committee (code 2012-025). All participants gave their written informed consent for data collection, and their provided data were completely anonymized. The study was performed in accordance with the Helsinki Declaration.

### Measures

The following battery of paper-and-pencil self-report measures was administered to the participants along with a sociodemographic questionnaire asking about age, gender, marital status, level of education, and employment.

The Goldberg Anxiety and Depression Scale (GADS; Goldberg et al., 1988) is an 18-item questionnaire that contains 2 subscales (i.e., Anxiety and Depression), each composed of 9 binary items (yes/no). The aim of this scale is to detect “probable cases”, orienting the clinician in the diagnosis (Montón et al., 1993). Scores for each subscale range from 0 to 9, where higher scores indicate more severity. Previous studies have proposed cut-off points (≥4 for anxiety and ≥2 for depression) for considering “probable cases” (Reivan-Ortiz et al., 2019). The Spanish version of the GADS presented 82% of specificity and 83% of sensibility, next to an adequate concurrent validity (Montón et al., 1993).

The Mindful Attention Awareness Scale (MAAS; Brown & Ryan, 2003) is a 15-item scale which measures mindfulness as a trait. Each item is scored using a 6-point Likert scale. The total score is calculated by the mean of the 15 items and ranges from 1 to 6, with higher scores indicating greater levels of mindfulness. The Spanish version of the MAAS (Soler et al., 2012) has shown appropriate convergent validity with other mindfulness measures, internal consistency (*α* = .89), and test-retest reliability (*r* = .82).

The Self-Compassion Scale-short form (SCS-SF; Raes et al., 2011) is a 12-item questionnaire designed to assess overall self-compassion and three dimensions: Common humanity, Mindfulness, and Self-kindness. For the present study only the total score, which is calculated by the mean of the 12 items, was considered (Neff et al., 2019). Total scores range from 1 to 5, with higher ones indicating greater levels of self-compassion. The Spanish version of the SCS-12 (Garcia-Campayo et al., 2014) has shown good internal consistency (*α* = .86) and very high convergence with the long form (26-items version) of the scale (*r* ≥ .97).

The Connor-Davidson Resilience Scale (CD-RISC; Campbell-Sills & Stein, 2007) consists of 10 items measuring resilience. Each item is scored in a 5-point Likert scale, and the total score, which ranges 1-5, is calculated by averaging the scores of the items; higher scores indicate greater resilience. The CD-RISC has shown good internal consistency (*α* = .89) and test-retest reliability (*r* = .87) in people with anxiety or stress-related disorders. The Spanish version (Soler Sánchez et al., 2016) presented good internal consistency (*α* = .86) and test-retest reliability (*r* = .87).

### Statistical analysis

**Figure 1:**
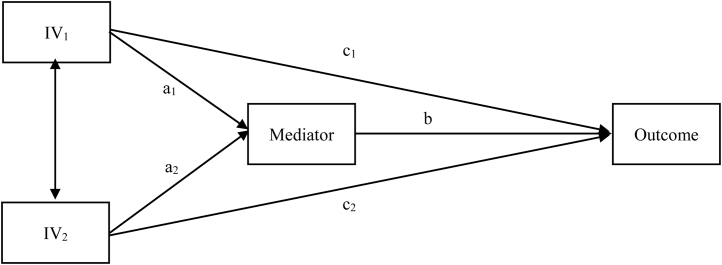
Generic example of a path analysis model with two correlated independent variables (IV) and one mediator.

Descriptive data analyses were performed for describing the sample, reporting frequencies and percentages for categorical data and means and standard deviations for continuous variables. Bivariate analyses were carried out by calculating correlation between variables, using Pearson’s φ coefficient, the point-biserial correlation (*r*_b_), and Pearson’s *r* coefficient when appropriate. Path analysis models were performed to test the study hypothesis; path analysis includes mediation effects and simultaneous estimation of the relationships among variables in order to estimate these relationships in an unbiased way (Lockhart et al., 2011, MacKinnon, 2008). Figure 1 shows a generic path analytic model with two (correlated) independent variables and one mediator. For our study, we computed two models considering MAAS and SCS-12 as independent variables, CD-RISC as a mediator, and GADS subscales as outcomes (i.e. probable case vs. improbable case of anxiety/depressive disorder using the abovementioned cut-off criteria). Standardized regression coefficients (β) of bias-corrected bootstrapped indirect effects based on 10,000 bootstrap samples were calculated as well as their standard error and 95% confidence interval (*CI*). Parameters of indirect effects were considered statistically significant when the 95% *CI* did not include 0 (Lockhart et al., 2011). The statistical packages used for the present study were SPSS v27.0 and Mplus v8.4.

## Results

### Descriptive analysis

The study sample was composed of 860 participants, among which 489 (56.90%) were women. Age ranged from 18 to 88, with a mean of 48.90 years (*SD* = 16.91). The age distribution was normal and similar to the Spanish population’s (Kolmogorov-Smirnov test’s *p* = .052). Most of the participants were married (*n* = 496, 57.70%) and had at least primary studies (*n* = 723, 84.10%). Almost half of them were employed (*n* = 378, 44%). The GADS-Anxiety presented a mean score of 1.60 (*SD* = 2.50), and the GADS-Depression a score of 1.14 (*SD* = 2.11). Considering the cutoff points mentioned above, 192 participants (22.30%) were “probable cases” of anxiety disorders, and 214 (24.90%) were “probable cases” of depression. These results are summarized in Table 1.

**Table 1:** Demographic and clinical characteristics of the sample.

	Total sample (*N* = 860)
Demographic characteristics	
- Sex, *n* women (%)	489 (56.90%)
- Age, M (SD)	48.87 (16.91)
○ 18-29	102 (11.90%)
○ 30-39	144 (16.70%)
○ 40-49	172 (20%)
○ 50-59	164 (19.10%)
○ 60-69	142 (16.50%)
○ 70-79	87 (10.10%)
○ ≥80	49 (5.70%)
- Marital status, *n* of married (%)	496 (57.70%)
- Educational status, *n* of university studies (%)	129 (14.90%)
- Employment status, *n* of currently working (%)	378 (44%)
Clinical characteristics [range]	
- GADS-Anxiety, *M* (*SD*) [0-9]	1.60 (2.50)
Probable cases, *n* (%)	192 (22.3%)
- GADS-Depression, *M* (*SD*) [0-9]	1.14 (2.11)
Probable cases, *n* (%)	213 (24.9%)
- MAAS, *M* (*SD*) [1-6]	4.51 (0.86)
- SCS-12, *M* (*SD*) [1-5]	3.16 (0.67)
- CD-RISC, *M* (*SD*) [1-5]	3.67 (0.73)

*Note.* GADS: Goldberg Anxiety and Depression Scale; MAAS: Mindful Attention Awareness Scale; SCS-12: Self-Compassion Scale, Short form; CD-RISC: Connor-Davidson Resilience Scale.

### Bivariate analysis

GADS-anxiety and GADS-depression were the most associated variables (see Table 2; Pearson’s φ = .51, *p* < .001). Significant moderately low and negative correlations were found between the GADS subscales and the other study measures, with *r*_b_ ranging between -.22 and -.32. MAAS, SCS-12 and CD-RISC presented moderate relationships, with *r* ranging from .31 to .48. All correlations remained significant (*p* < .001) when controlling for CD-RISC.

**Table 2:** Correlations between psychological variables. Between brackets, partial correlations controlling for CD-RISC.

	GADS-Depression	MAAS	SCS-12	CD-RISC
GADS-Anxiety	.51* (.49*)	-.30* (-.22*)	-.31* (-.21*)	-.22*
GADS-Depression		-.32* (-.24*)	-.31* (-.19*)	-.25*
MAAS			.37* (.27*)	.31*
SCS-12				.48*

*Note.* * means *p* value <.001.

### Path analysis for probable cases of anxiety disorders

**Figure 2:**
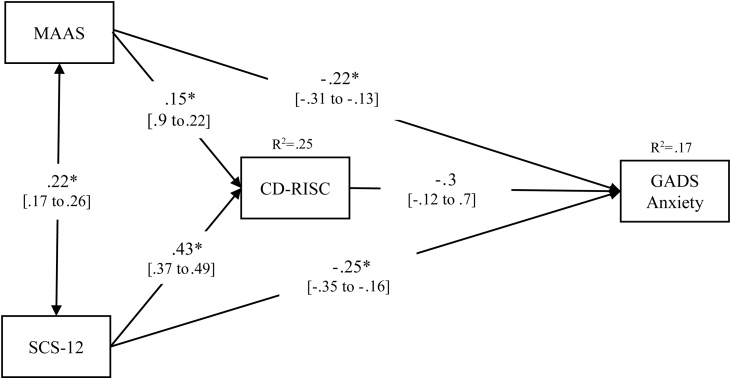
Path analysis model for GADS-anxiety. *Note.* * means *p* value < .001; *R^2^* represents the proportion of the variance for a dependent variable that is explained by an independent variable or variables. All coefficients are standardized. 95% CI are detailed between brackets.

The path analysis model showed significant direct effects of the two independent variables on the outcome, but no significant effect was found of the mediator on the outcome (*p* = .619) and, therefore, the indirect effects were not statistically significant. Table 3 presents the unstandardized coefficients, standard errors, and the statistical significance of the direct and indirect effects, and the paths are represented in Figure 2 including the corresponding standardized coefficients.

**Table 3:** Direct and Bootstrap indirect effects in the multiple mediational models for GADS-anxiety.

Direct effects	Path	Coeff.	SE	*p* value
MAAS→CD-RISC	a_1_	1.30	0.30	<.001
MAAS→GADS-Anxiety	c_1_	-0.52	0.11	<.001
SCS-12→CD-RISC	a_2_	4.62	0.34	<.001
SCS-12→GADS-Anxiety	c_2_	-0.75	0.15	<.001
CD-RISC→GADS-Anxiety	b	-0.01	0.01	.619

Indirect effects	Path	Boots.	SE	95% CI
MAAS→CD-RISC→GADS-Anxiety	a_1_×b	-0.01	0.02	-0.05 to 0.03
SCS-12→CD-RISC→GADS-Anxiety	a_2_×b	-0.03	0.06	-0.15 to 0.09
Total indirect effects		-0.04	0.15	-0.20 to 0.12

*Note.* GADS = Goldberg Anxiety and Depression Scale; MAAS = Mindful Attention Awareness Scale; SCS-12=Self-Compassion Scale, Short form; CD-RISC = Connor-Davidson Resilience Scale. These are non-standardized results; standardized results are reported in Figure 2.

### Path analysis for probable cases of depression

**Figure 3:**
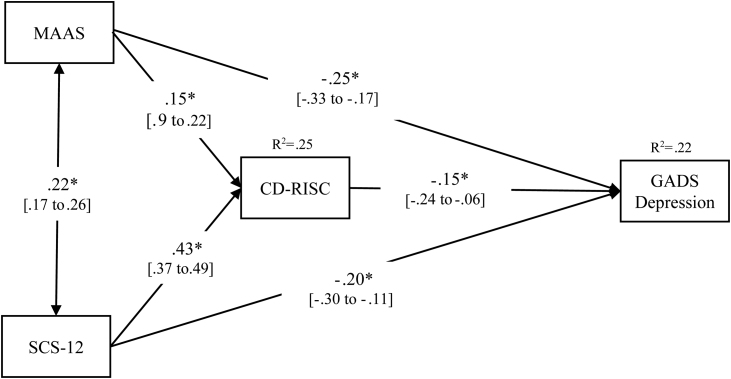
Path analysis model for GADS-depression. *Note.* * means *p* value < .001; *R^2^* represents the proportion of the variance for a dependent variable that is explained by an independent variable or variables. All coefficients are standardized. 95% CI are detailed between brackets.

A partial mediation effect of resilience was observed; the direct effects were statistically significant (all *p* values < .001), including the effect of CD-RISC on the outcome (*p* = .002), and the indirect effects were also statistically significant. Table 4 presents the unstandardized coefficients, standard errors, and the statistical significance of the direct and indirect effects, and the paths are represented in Figure 3 including the corresponding standardized coefficients.

**Table 4:** Direct and Bootstrap indirect effects in the multiple mediational models for GADS-depression.

Direct effects	Path	Coeff.	SE	*p* value
MAAS→CD-RISC	a_1_	1.30	0.30	<.001
MAAS→GADS-Depression	c_1_	-0.59	0.11	<.001
SCS-12→CD-RISC	a_2_	4.62	0.34	<.001
SCS-12→GADS-Depression	c_2_	-0.61	0.15	<.001
CD-RISC→GADS-Depression	b	-0.04	0.01	.002

Indirect effects	Path	Boots.	SE	95% CI
MAAS→CD-RISC→GADS-Depression	a_1_×b	-0.05	0.02	-0.11 to -0.02
SCS-12→CD-RISC→GADS-Depression	a_2_×b	-0.19	0.06	-0.33 to -0.07
Total indirect effects		-0.25	0.16	-0.41 to -0.09

*Note.* GADS = Goldberg Anxiety and Depression Scale; MAAS = Mindful Attention Awareness Scale; SCS-12=Self-Compassion Scale, Short form; CD-RISC = Connor-Davidson Resilience Scale. These are non-standardized results; standardized results are reported in Figure 3.

## Discussion

The present work aimed at exploring the intermediary role of resilience between ‘third-wave’-related constructs (mindfulness and self-compassion) and psychopathological symptoms (anxiety and depression). Our results bear out the expected relationships between the variables, considering previous works: mindfulness presented significant positive associations with self-compassion (Baer et al., 2012) and resilience (Kemper et al., 2015, Montero-Marin et al., 2015), and negative correlations with anxiety and depression (Takahashi et al., 2019). Similarly, self-compassion showed positive correlations with resilience (Kemper et al., 2015, Neff and McGehee, 2010) and was negatively associated with anxiety and depression (Mehr and Adams, 2016, Van Dam et al., 2011), although the effect sizes were smaller than expected. The results of the path analysis model supported our first hypothesis, as both mindfulness and self-compassion had significant direct effects on anxiety and depressive symptomatology; however, contrary to what was hypothesized following previous findings (Quist-Møller et al., 2018, Van Dam et al., 2011), self-compassion’s direct effect was not significantly stronger than mindfulness’.

The second hypothesis was not supported by our results in the case of anxiety symptomatology: resilience showed a non-significant effect on anxiety after controlling for mindfulness and self-compassion, which implied that the indirect paths were not statistically significant. Thus, and contrary to expectations, resilience was not a significant mediator of the effect of mindfulness and self-compassion on anxiety symptomatology in our sample. Although resilience had been identified as a mediator of the effect of mindfulness, it was on other outcomes such as emotion regulation (Wang et al., 2016) and positive affect (Bajaj & Pande, 2016), which can be related to anxiety symptoms, but would probably be more directly associated to depression. Previous works have observed that other ‘third-wave’ constructs mediate the effect of mindfulness on anxiety, such as non-attachment (Whitehead et al., 2019), which is defined as freedom from unhealthy cognitive fixations on objects and others (Deits-Lebehn et al., 2019), and also decentering, defined as the capacity to observe items that arise in the mind as mere psychological events (Hoge et al., 2015). Further studies should replicate these findings and include other ‘third-wave’ core constructs, such as psychological flexibility, which has been found to mediate effects of ‘third-wave’ psychotherapies on outcomes such as stress and anxiety in clinical samples (Montero-Marín et al., 2018, Pérez-Aranda et al., 2019, Wicksell et al., 2010) and is defined as the ability to feel and think with an open mind while forging habits that allow us to live in a way that is consistent with our values and aspirations (Hayes, 2020; page 17).

On the other hand, our hypothesis was partially supported in the case of depressive symptomatology: both direct and indirect paths were significant. That implies a possible partial mediation of resilience that goes in line with the results reported by previous studies: Wang et al. (2016) found that emotional resilience partially mediated the effect of mindfulness on emotion regulation in college students in China; similarly, Bajaj and Pande (2016) reported a partial mediation effect of resilience on mindfulness effect on life satisfaction, positive and negative affect in university students in India. In this regard, it is noteworthy that some authors consider emotional resilience to be a potential mechanism of mindfulness (Hayes and Feldman, 2006, Polizzi et al., 2018); mindfulness may promote the generation of positive emotions and the ability to recover from negative emotions, helping the individual to maintain a decentered attitude toward difficult situations (Bajaj & Pande, 2016). These mechanisms would help the person to regulate emotions, increase life satisfaction and reduce negative affect (Wang et al., 2016), making them less likely to present depressive symptomatology.

For what concerns to self-compassion, no other work has studied how its effect on anxiety and depression could be mediated by resilience; however, different studies have reported significant associations between resilience and self-compassion (Kemper et al., 2015, Neff and McGehee, 2010), and considering its tight relation with mindfulness (Neff, 2003), it could be expected that similar mechanisms–i.e. generating positive emotions, in this case through the desire to alleviate one’s suffering and to heal oneself with kindness–could explain the impact of self-compassion on depression through the mediating effect of psychological resilience.

Some limitations of this work must be acknowledged; first, the cross-sectional design of this study cannot determine a causal relationship, so the results are exploratory and should be interpreted with caution. Second, despite using a large sample of Spanish general population individuals, all of them were from the same region and, therefore, the results could not be completely representative of the Spanish population. Third, the use of self-reported measures in this study, particularly the GADS, undermines the strength of the outcomes, as it is only an indicator of “probable cases” of anxiety disorders and depression, and previous studies have identified specificity and sensitivity issues with this scale. It is likely that the use of standardized psychiatric interviews (SCID, CIDI, MINI, etc.) conducted by trained interviewers would have yielded a lower prevalence of anxiety and depression cases. Fourth, this study considers mindfulness as a unidimensional trait, but there is consistent evidence that it could be a multi-facet construct and, thus, future studies should replicate our work examining the role of the different facets of mindfulness. Finally, it needs to be considered that the data were collected between 2012 and 2015, which implies that the results should be framed in the socioeconomic context of that period; the impact of the global economic crisis on mental health was particularly noteworthy in Spain (Bartoll et al., 2014, Gili et al., 2013), which could justify the high proportion of “probable cases” of anxiety and depressive disorders among our sample. Thus, future studies should replicate our model using more recent data and considering other designs such as longitudinal studies.

## Conclusions

The results of the present study suggest that partial mediation of resilience is playing a significant role on the effect of mindfulness and self-compassion on depression, but not on anxiety symptoms. These latter could be mediated by other ‘third-wave’ variables such as decentering, non-attachment or psychological flexibility, as previous studies have suggested.
